# *HERMES* – a GUI-based software tool for pre-processing of X-ray absorption spectroscopy data from laboratory Rowland circle spectrometers

**DOI:** 10.1107/S1600577521012583

**Published:** 2022-01-01

**Authors:** Marco E. Seddon-Ferretti, Lucy M. Mottram, Martin C. Stennett, Claire L. Corkhill, Neil C. Hyatt

**Affiliations:** a Department of Materials Science and Engineering, University of Sheffield, Mappin Street, Sheffield S1 3JD, United Kingdom; b Department of Computer Science, University of Sheffield, Regent Court, Sheffield S1 4DP, United Kingdom

**Keywords:** XAS, XANES, XAFS, laboratory spectrometer, data processing

## Abstract

*HERMES*, a graphical user interface software tool, is presented, for pre-processing X-ray absorption spectroscopy (XAS) data from laboratory Rowland circle spectrometers, to meet the data handling needs of a growing community of practice. *HERMES* enables laboratory XAS data to be displayed for quality assessment, merging of data sets, polynomial fitting of smoothly varying data, and correction of data to the true energy scale and for dead-time and leakage effects. The software is written in Java 15 programming language, and runs on major computer operating systems, with graphics implementation using the JFreeChart toolkit. *HERMES* is freely available and distributed under an open source licence.

## Abstract 2

*HERMES* is a graphical user interface software tool for pre-processing X-ray absorption spectroscopy data from laboratory Rowland circle spectrometers.

## 1. Introduction

The renaissance of laboratory X-ray absorption spectroscopy (XAS) instrumentation is revolutionizing access to, and uptake of, this technique across the physical sciences and engineering, enabling application of this technique without the need for access to a synchrotron light source (Błachucki *et al.*, 2019 ▸; Honkanen *et al.*, 2019 ▸; Jahrman *et al.*, 2019*a* ▸; Schlesiger *et al.*, 2015 ▸; Malzer *et al.*, 2018 ▸; Mortensen *et al.*, 2016 ▸; Németh *et al.*, 2016 ▸; Seidler *et al.*, 2014 ▸, 2016 ▸; Zeeshan *et al.*, 2019 ▸). In particular, commercial and user-built instrumentation based on a Rowland circle spectrometer with spherically bent crystal analyzers (SBCAs) used in the Johann configuration, and utilizing an energy-dispersive X-ray (EDX) detector, are gaining adoption, both as laboratory and regional facilities with a role complementary to, and symbiotic with, use of synchrotron radiation sources (Ditter *et al.*, 2019 ▸). Already, this spectrometer design has been exploited to address a wide range of problems in nuclear, functional, catalysis and geological materials, including operando studies (Bès *et al.*, 2018 ▸; Bi *et al.*, 2019*a* ▸,*b* ▸; Jahrman *et al.*, 2019 ▸ *b*; Kuai *et al.*, 2018 ▸; Lutz & Fittschen, 2020 ▸; Mottram *et al.*, 2020*a* ▸,*b* ▸,*c* ▸; Moya-Cancino *et al.*, 2019 ▸; Nolis *et al.*, 2020 ▸; Sun *et al.*, 2021 ▸; Wittkowski *et al.*, 2021 ▸; Zimmermann *et al.*, 2021 ▸).

The rapid uptake of the Rowland circle XAS spectrometer is driving an expansion of the user base for the technique, who require tools to integrate and pre-process data for further analysis. This need arises because, typically, several scans are acquired with (*I*_t_) and without (*I*_0_) the sample, to compute the absorption; whereas, at a synchrotron source, *I*_0_ and *I*_t_ would be acquired simultaneously. Recently, Bès *et al.* (2018 ▸, 2021 ▸) have demonstrated an elegant procedure for acquisition of *I*_0_ and *I*_t_ simultaneously, by exploiting SBCA harmonics, although this is not always applicable. Raw data also need to be quality assessed, dead-time corrected, appropriately merged, and corrected to the true energy scale and for leakage effects. Although data processing codes have been developed within Jupyter notebook and Mathematica environments, they require some familiarity with coding to use efficiently and troubleshoot problems. However, this may not necessarily be within the grasp of a broad user base, for whom XAS is a supplementary or infrequent analytical tool. We therefore developed *HERMES* as software based on an intuitive graphical user interface (GUI), to enable rapid and robust pre-processing of laboratory XAS data from Rowland circle spectrometers, for import into software such as *ATHENA* for further analysis (Ravel & Newville, 2005 ▸). Subsequently, the *HERMES* backronym was later coined – Handy Energy Recalibration and Mu Evaluation Software.

*HERMES* is free to download and distributed under an Open Source Initiative approved MIT Licence (Open Source Imitative, 2021 ▸), and documentation is distributed under a Creative Commons CC BY 4.0 licence (Creative Commons, 2021 ▸), enabling users to adapt and modify the source code to better meet their needs, as may be desirable. *HERMES* is written in Java 15 and compiled and tested to work on the common laboratory Microsoft Windows and Macintosh OSX platforms. Plotting graphics are implemented using JFreeChart (JFreechart, 2021 ▸). Java was chosen for implementation due to its strong object orientation and type safety.

## 2. Features of *HERMES*

*HERMES* is a program for pre-processing of transmission mode laboratory X-ray absorption spectroscopy data, from Rowland circle spectrometers, to produce input files suitable for further analysis using software such as *ATHENA*. It provides the following functionality:

(i) Dead-time correction of raw data.

(ii) Plotting and comparison of multiple *I*_0_ and *I*_t_ data.

(iii) Fitting of a polynomial to suitable *I*_0_ and *I*_t_ data.

(iv) Merging of several *I*_0_ and *I*_t_ data sets.

(v) Evaluation of absorption μ(*E*) from *I*_0_ and *I*_t_ data.

(vi) Correction of data for leakage effects.

(vii) Recalibration of data energy scale.

**Figure 1:**
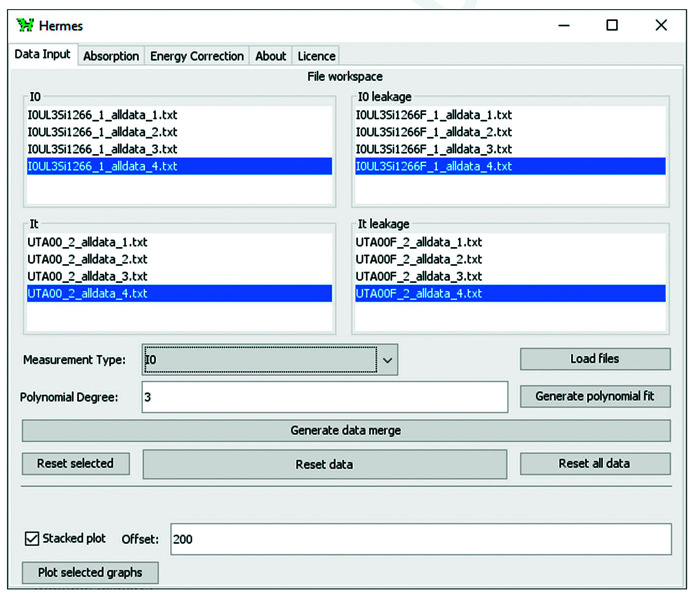
*HERMES* dashboard showing the Data Input tab, with four file workspaces, measurement type dropdown menu, data processing and plotting tools.

**Figure 2:**
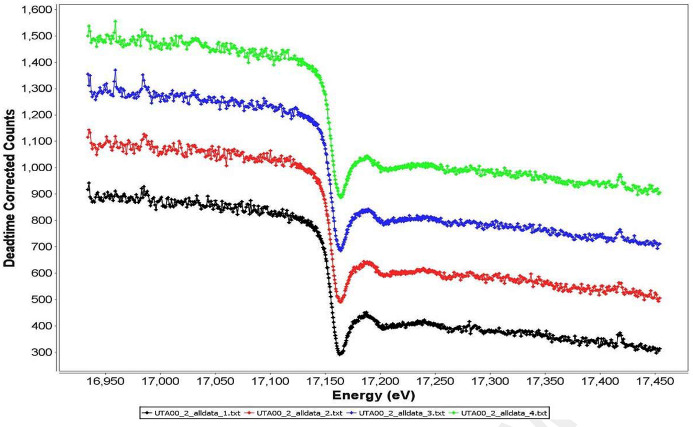
Example of the *HERMES* plotting window, showing a stack plot of *I*_t_ data at the U *L*_3_-edge, separated by a user-specified offset.

*HERMES* uses a logical workflow to guide the user through the steps of data pre-processing. The dashboard has a simple and intuitive interface, with four data workspaces and processing tools, shown in Fig. 1 ▸. The user specifies the Measurement Type to be loaded or processed using a dropdown menu (*I*_0_, *I*_t_, *I*_0_ leakage, *I*_t_ leakage). The user is required to select appropriate columns for energy, theta, detector raw counts, detector input count rate (ICR), and detector output count rate (OCR). A first-order dead-time correction is applied to raw detector counts, valid for dead-time up to 50% (XIA LLX, 2009 ▸). The plotting function supports enlargement of regions of interest and data may be displayed individually, overlaid or offset (by a user-specified amount), as shown in Fig. 2 ▸. An *n*th-order polynomial (where *n* is user specified) may be fitted to any appropriate and smoothly varying data set selected (*i.e.* *I*_0_, *I*_0,lk_, *I*_t,lk_; where lk denotes a leakage measurement, as discussed below).

After the user has completed plotting, assessment, merging and polynomial fitting of raw data in the first tab of the dashboard, the workflow progresses naturally to the second tab where the user may evaluate and inspect the absorption spectrum, μ(*E*). If the specimen is sufficiently thick and/or attenuating it may be desirable to correct the computed absorption data for ‘leakage effects’ (Stern & Kim, 1981 ▸; Mottram *et al.*, 2020*a* ▸) which may arise from contamination of the transmission data by harmonics, stray scatter and the low energy tail of the monochromator function; this correction is effected by a tick box. In the Rowland circle geometry, it may be necessary to measure transmission data with a large detector offset (*I*_0,lk_ and *I*_t_,_lk_), to correct the absorption data for distortion arising from leakage effects according to equation (1),

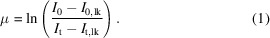

Where leakage effects are not important, then *I*_0,lk_ = *I*_t,lk_ = 0. The fitted polynomials, without Poisson noise, may be used to evaluate the absorption, if desirable and appropriate.

Evaluation of absorption requires the user to specify one data set each of *I*_0_ and *I*_t_ (plus *I*_0,lk_ and *I*_t,lk_, if required), which may be raw, merged or polynomial fitted data. The absorption is displayed as a function of energy and theta. The computed absorption, merged, and polynomial fit data are written as text files, at the point of computation, together with a list file to enable data provenance and curation.

The third tab in the *HERMES* workflow enables calibration of the absolute energy scale of absorption spectra evaluated in the previous workspace. In general, the absolute energy scale of laboratory XAS data requires calibration using a suitable reference material, for which there are calibrated literature, open source or user-acquired XAS data. Rowland circle spectrometers function on an angle-dispersive principle to maintain the required focusing condition. Steps in energy or *k*-space, within user-specified ranges, determine the required steps in theta space according to the Bragg Law,

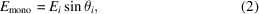

where *E*_mono_ is the characteristic backscatter energy of the SBCA. *HERMES* determines *E*_mono_ from a user-specified absorption spectrum, within the workspace. Since the relationship between energy and theta is non-linear, it is necessary to apply the correction in theta space and then recalibrate the energy scale. *HERMES* requires the user to specify the observed and true energy (*E*_obs_ and *E*_true_) of some feature in the absorption spectum, such as the maximum in the first derivative of μ(*E*). From equation (2), the corresponding θ_obs_ and θ_true_ are determined, the difference between these values being the Δθ_shift_ required to align the absorption spectrum in theta space. The absorption spectrum is calibrated by applying the theta shift to the observed data and calculation of the true energy, from equation (2). The original and calibrated absorption spectrum are plotted in energy and theta space for inspection, post calibration. The calibrated absorption spectrum is written as a text file, at the point of computation, with both original and calibrated energy and theta scales, together with a list file detailing the key calibration parameters.

A comprehensive user guide and video tutorial are provided to support use of the software (see the supporting information).

## 3. Conclusions

We have presented the *HERMES* software for pre-processing of laboratory X-ray absorption spectroscopy data from Rowland circle spectrometers. A simple GUI and intuitive workflow enable integration, correction and calibration of raw data to output data files suitable for further analysis in software such as *ATHENA*. This software contributes to meeting the need of a rapidly growing community of practitioners, who require freely available tools for rapid and robust pre-processing of laboratory XAS data.

## 4. Resources

A project page for *HERMES* exists at https://github.com/xasheffield/hermes. *HERMES* is available as an executable .jar file (Windows, MacOS, requiring an existing installation of Java) or as a .exe file with the necessary Java Runtime bundled (Windows only), and both are freely available at the link above, as well as a complete user manual; a tutorial video is available (see the supporting information).

## Supplementary Material

HERMES user guide. DOI: 10.1107/S1600577521012583/ok5064sup1.pdf

HERMES video tutorial. DOI: 10.1107/S1600577521012583/ok5064sup2.mp4
