# Supplementary material for: HERMES – a GUI-based software tool for pre-processing of X-ray absorption spectroscopy data from laboratory Rowland circle spectrometers
Source: J Synchrotron Radiat. 2022 Jan 1;29(Pt 1):276–9. doi: 10.1107/S1600577521012583 (PMC8733975; doi:10.1107/S1600577521012583)
Supplement: Supplementary file 1 [file s-29-00276-sup1.pdf]

HERMES USER GUIDE

Software for pre-processing of laboratory XAS data

Abstract

Introduction to the functionality and use of HERMES in pre-processing of laboratory XAS data – a.k.a. Handy Energy Recalibration and Mu Evaluation Software.

M.E. Seddon-Ferretti, L.M. Mottram, M.C. Stennett, N.C. Hyatt

University of Sheffield, 2021.

Version 1.0.0

This documentation is licensed under a Creative Commons CC BY 4.0 license.

HERMES - software for pre-processing of laboratory XAS data

> *"Zeus who masses the clouds made answer . . . turned to his dear son Hermes : 'Hermes, you are always our messenger.'"*
>
> **Homer, Odyssey 5. 4 ff.**

1. Introduction

HERMES is a GUI based utility for rapid and robust pre-processing of transmission laboratory XAS data from Rowland circle spectrometers [1-6], for import into software such as ATHENA for further analysis [7]. Following development, the HERMES backronym was later coined as Handy Energy Recalibration and Mu Evaluation Software.

HERMES is free to download and distributed under the MIT Licence approved by the Open Source Imitative [8], the documentation is distributed under Creative Commons CC BY 4.0 licence [9], enabling users to adapt and modify the source code to better meet their needs, as may be desirable. HERMES is written in Java 15 and compiled and tested to work on the common laboratory Microsoft Windows and Macintosh OSX platforms. Plotting graphics are implemented using JFreeChart [10]. Java was chosen for implementation due to its strong object orientation and type safety. The software is supplied without warranty against harm or damage to the computer system of the user. Users are advised to ensure that their Java version is fully up to date to avoid problems in functionality and stability.

2. Development and citation

HERMES was developed at the University of Sheffield, by Prof. Neil Hyatt, Ms. Lucy Mottram, Dr Martin Stennett and Mr. Marco Seddon-Ferretti, with financial support from UK Research and Innovation. The software was coded by Marco and tested by Lucy, Martin and Neil. Please cite the software as: HERMES - software for pre-processing of laboratory XAS data, M.E. Seddon-Ferretti, L.M. Mottram, M.C. Stennett, and N.C. Hyatt, 2021.

3. Features of Hermes

Hermes provides the following functionality:

- Deadtime correction of raw detector data
- Plotting and comparison of multiple I_0 and I_t data
- Fitting of a nth order polynomial to suitable I_0 and I_t data
- Merging of several I_0 and I_t data
- Evaluation of absorption μ(E) from I_0 and I_t data
- Correction of data for leakage effects
- Recalibration of the data energy scale

The plotting function supports enlargement of regions of interest and data may be displayed individually, overlaid or offset (by a user specified amount).

4. The HERMES dashboard

To launch HERMES, double click on its icon, which will initiate the HERMES dashboard shown in Figure 1. The dashboard uses a logical workflow to guide the user through the

steps of data pre-processing, by sequentially working through the Data Input, Absorption and Energy Correction tabs. Hovering the pointer over each tool will display a brief description.

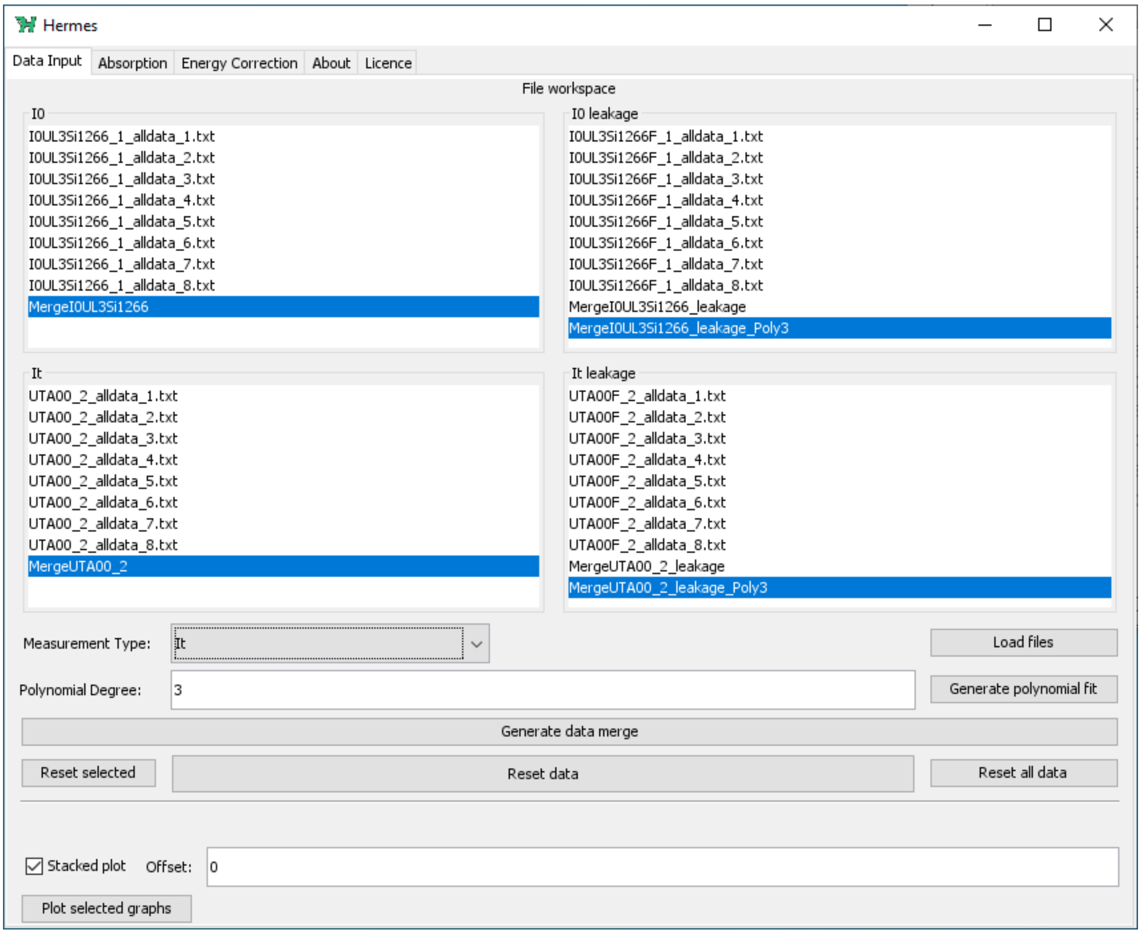

Figure 1: HERMES dashboard showing the Data Input tab, with four file workspaces, measurement type dropdown menu, data processing and plotting tools.

5. The Data Input tab

Within this tab are four workspaces for data input and tools for merging, polynomial fitting and plotting of data; see Figure 1.

5.1 Setting the data type to load, process or plot

The **Measurement type** drop down menu selects the data type to be loaded or processed, either I_0, I_t, I_0 leakage or I_t leakage. Note that actions will be applied to the measurement type designated in this drop down menu. For example, if the user selects I_t in this menu, then clicking **Load files** or **Generate data merge** will input or operate on data within the I_t workspace; see Figure 1.

5.2 Load files

To load data files for plotting and processing, first select the **Measurement type**, then click **Load files**, and navigate to the folder containing the data. HERMES assumes data are in text file format, as tab separated entries, with column headers beginning below a separating line defined by an asterisk (*). When the first data are loaded, a dialogue box asks the user to specify the columns corresponding to Energy (eV), Theta (degrees), detector Counts, Input Count Rate – ICR, and Output Count Rates – OCR. This is achieved by using the **drop down menus** as shown in Figure 2; *if a mistake is made in this step, simply close and*

restart the program. Subsequent data are assumed to have the same format and be of the same length (if this is not the case, an error message will flag during processing). Figure 1 shows the example data supplied with HERMES loaded into the four workspaces. Remember to change the measurement type using the drop down menu according to the file space when plotting or processing data!

HERMES reads the file header information before the separating line defined by an asterisk; the information may extend over more than one line. When a merged or Absorption File is saved, the header information and original file name are written to an associated list file to assist data provenance and curation.

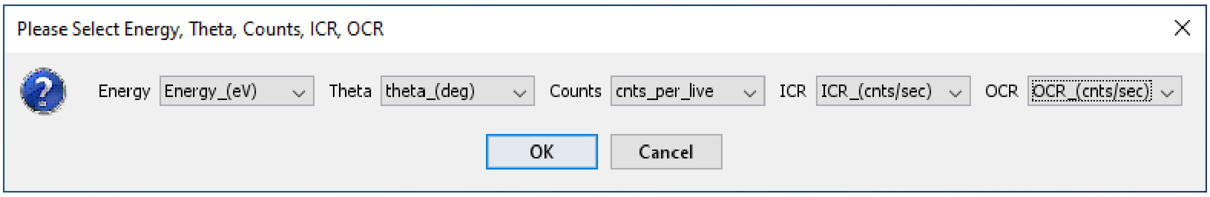

Figure 2: Dialogue box enabling the user to map Energy (eV), Theta (degrees), detector Counts, Input Count Rate, and Output Count Rate, to column headings in data file format using drop down menus; here the drop down menu maps Counts to the cnts_per_live channel of the detector.

5.2 Deadtime correction

HERMES Version 2 automatically performs a first order deadtime correction to the measured detector counts. The measured detector counts (N_i) and corrected detector counts ($N_{corr,i}$) are related by Equation 1, where ICR_i and OCR_i are the associated Input Count Rate and Output Count Rate:

$$N_{corr,i} = N_i \left(\frac{ICR_i}{OCRi}\right) \qquad \text{Eq. 1}$$

This correction is valid only where the OCR values are less than 50% of the maximum OCR value and a more complex approach is required, which is not currently implemented in HERMES [11]. Data may be processed without a deadtime correction using Hermes Version 0, if desirable.

5.3 Reset selected data

If the wrong data are loaded into a workspace (e.g. I_0 data mistakenly loaded into the I_t workspace), click to highlight the file(s) in the workspace concerned and then click **Reset selected**. Clicking “Yes” in the dialogue box will clear the selected data in the workspace to allow the correct data to be loaded.

5.4 Reset all data

To clear *all data* in *all workspaces* click **Reset all data** and “Yes” in the subsequent dialogue box.

5.5 Plot graphs

To display data loaded into the workspaces, first choose the **Measurement type**. Select the data to be plotted within the designated workspace by clicking to highlight the data file, hold shift to select data files in sequence, or hold control to select non-sequential data files. To plot the data click **Plot selected graphs**. The selected data will be displayed as individual

plots of Deadtime Corrected Counts vs. Energy (eV), see Figure 3. This mode of plotting is useful for initial survey of the data to identify obvious problems, such as a detector fault.

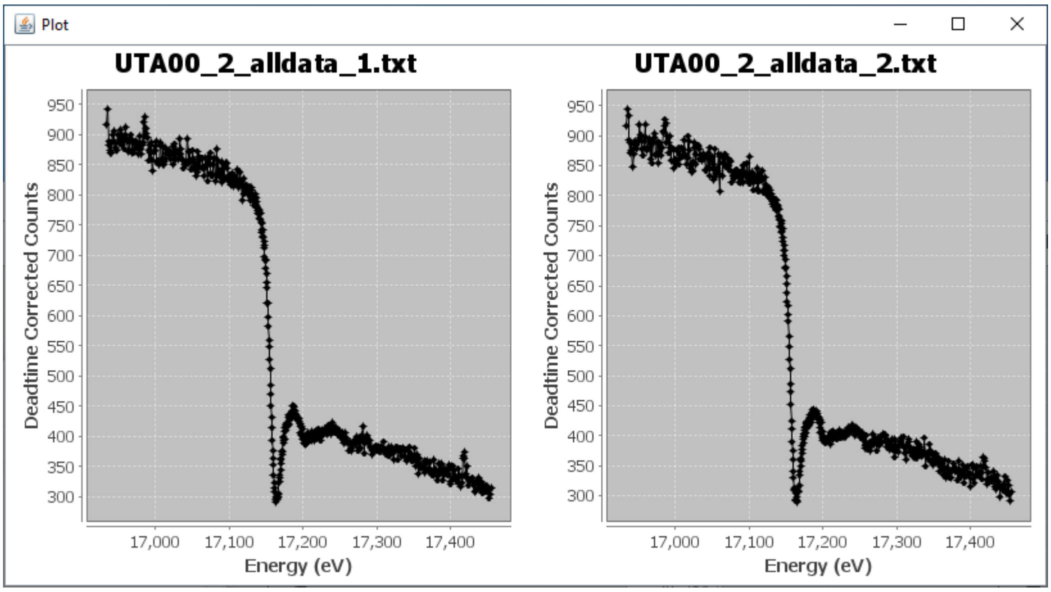

Figure 3: Example of two independent I_t data files displayed in separate panels using **Plot selected graphs**; the **Stacked Plot** box is unchecked.

Right clicking in a plot presents a menu for operating on the plot with the following functionality, see Figure 4:

- Properties: change the presentation of the plot.
- Copy: copies the panel as a PNG graphic.
- Save as…: saves the panel as a .PNG graphic file.
- Print…: prints the panel.
- Zoom in: allows magnification of the plot.
- Zoom out: allows zoom in of the plot.
- Zoom out: allows zoom out of the plot.
- Auto-range: allows the magnification / zoom to be reset.

A region of interest may be examined in any individual plot by clicking in the plot and dragging a box to magnify the select region, see Figure 4. This method is preferred over using the Zoom in / out functionality in the menu summarised above. To reset the zoom / magnification it is recommended to right click on the plot, choose “Auto Range” in the menu, then “Both Axes”, as shown in Figure 4. Alternatively, to zoom out click and drag left in the plot window.

All plot windows can be maximised, minimised and resized in the normal way, they remain visible until being closed in the normal way.

Data may also be displayed as an overlay or stack plot, which is preferred for comparison of data. This feature is implemented by checking the **Stacked plot** box. In the adjacent **text box** enter a value for the **Offset** on the y-axis. Clicking **Plot selected graphs** will now produce a stack plot of the data with each data set offset on the y-axis by the user specified amount; see Figure 5. An overlay plot may be produced by checking the **Stacked plot** box and setting the **Offset** on the y-axis to zero; see Figure 6. This visualisation is particularly useful for checking data alignment on the energy scale.

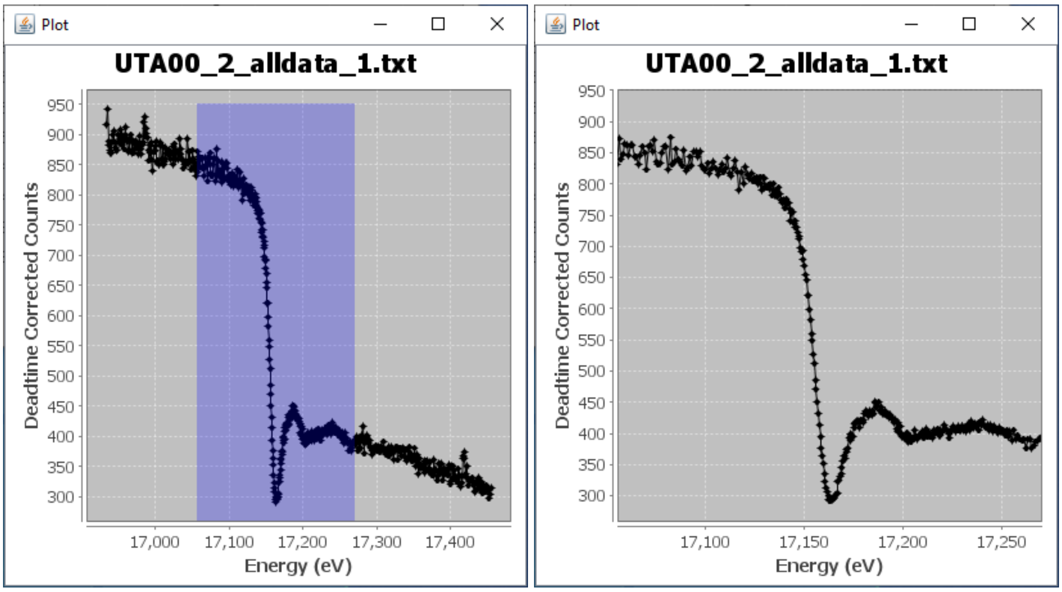

Figure 4: Example of: left) selecting a region of interest using a mouse to drag a box; and right) resulting zoomed region of interest, with plot functionality menu shown as activated by right click in the plot.

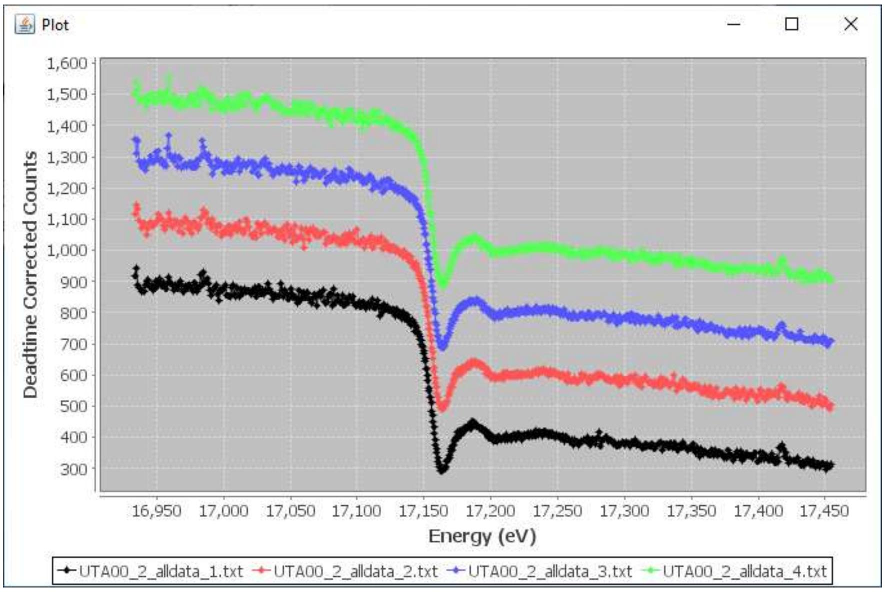

Figure 5: Example of the same four independent I_t data files shown in Figure 2, now displayed as a stack plot using **Plot selected graphs**; the **Stacked plot** box is checked and the **Offset** was specified in the text box as 200.

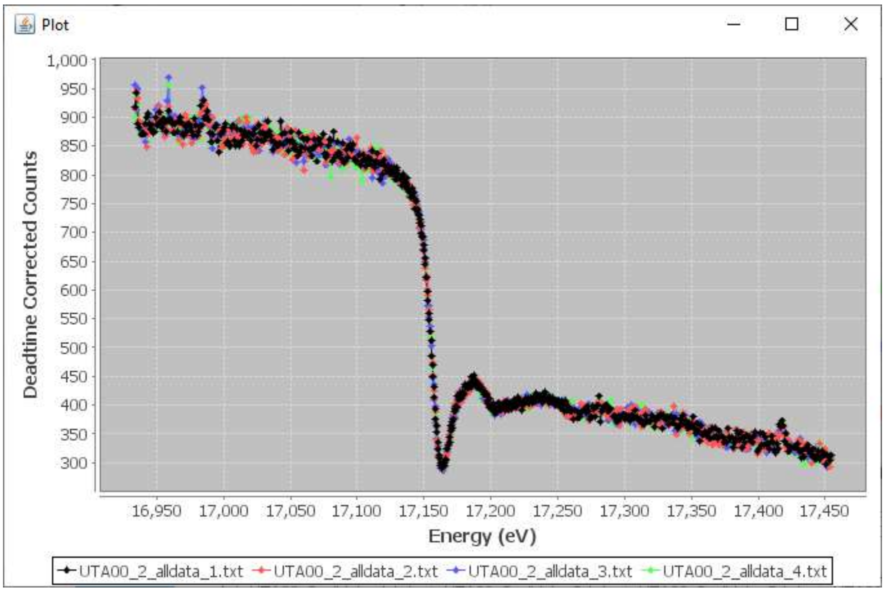

Figure 6: Example of the same four independent I_t data files shown in Figure 2, now displayed as an overlay plot using **Plot selected graphs**; the **Stacked plot** box is checked and the **Offset** was specified in the text box as zero.

5.6 Generate data merge

Several individual data files may be averaged to improve the signal to noise ratio in a resultant merged data set. Only data with the same range can be merged and each data file is assigned the same weighting factor. Prior to merging data it is recommended that an overlay plot is generated to determine that the data are well aligned on the energy scale. Should this not be the case, the ATHENA program provides tools for data alignment.

To merge data sets, first choose the **Measurement type**. Select the data to be merged within the designated workspace by clicking to highlight the data files. Click the **Generate data merge** button to merge the data. Enter a filename and header for the new merged data file in the dialogue boxes. The header can contain any information useful to the user, for example, relating to the sample composition or parameters of measurement. The merged data file and an associated list file are written to the same folder as the original data, and are automatically loaded into the workspace. If the data to be merged are not of the same range, the user will be notified with an error message and HERMES will truncate the output data file to the range of the shortest input file. The merged data can be plotted in the normal way as described above.

5.7 Generate polynomial

A polynomial of order *n* may be fitted to any appropriate and smoothly varying data set. A fitted polynomial has the benefit of not contributing Poisson noise if it is used instead of data in evaluation of the absorption. Polynomial fitting may be performed on an individual or merged data set.

First choose the **Measurement type** to be fitted with a polynomial. Select the data to be fitted within the designated workspace by clicking to highlight the data file. Enter the polynomial degree in the text box, usually a third order polynomial will suffice. Click the **Generate polynomial fit** button to fit the data. Enter a filename for the new polynomial fit data file in the dialogue boxes. The polynomial fit file is written to the same folder as the original data and are automatically loaded into the workspace; the polynomial coefficients

are written to the header of the polynomial fit file. The polynomial fitted data can be plotted in the normal way as described above, see Figure 7 as an example.

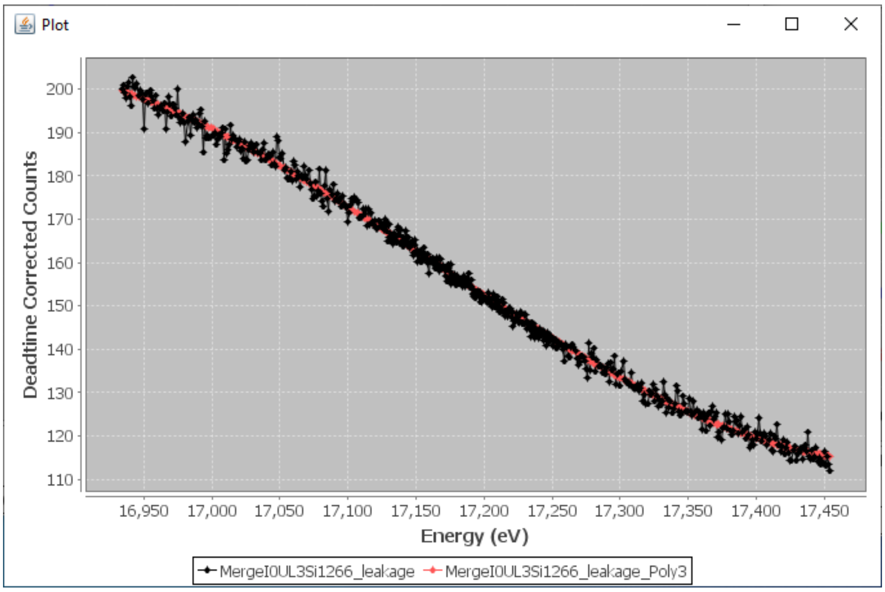

Figure 7: Example of a third order polynomial fit (red line) to merged I_0 leakage data (black points), displayed as an overlay plot using **Plot selected graphs**; the **Stacked plot** box is checked and the **Offset** was specified in the text box as zero.

6. The Absorption tab

Within this tab there are either two or four workspaces for data from which to evaluate the absorption, as shown in Figure 8. The data sets are pulled through from the **Data input tab** and must be input as described above. The default presentation of the dashboard shows two workspaces, one each for I_0 and I_t; if a significant leakage correction is required, checking the **Leakage is significant** box will add two more workspaces for the measured leakage data, one each for I_0 leakage and I_t leakage.

If the specimen is sufficiently thick and/or attenuating it may be desirable to correct the computed absorption data for leakage effects, which may arise from contamination of the transmission data by harmonics, stray scatter and the low energy tail of the monochromator function [12,13]; this correction is effected by a tick box. In such circumstances it is necessary to measure transmission data, using a large detector offset in Rowland circle geometry [13], with the sample ($I_{t,lk}$), and without the sample ($I_{0,lk}$), to correct the absorption data for distortion arising from leakage effects according to Equation 2:

$$\mu = ln\left(\frac{I_0 - I_{0,lk}}{I_t - I_{t,lk}}\right) \qquad \text{Eq. 2}$$

Where leakage effects are not important, then $I_{0,lk} = I_{t,lk} = 0$. The fitted polynomials, without Poisson noise, may be used to evaluate the absorption, if desirable and appropriate.

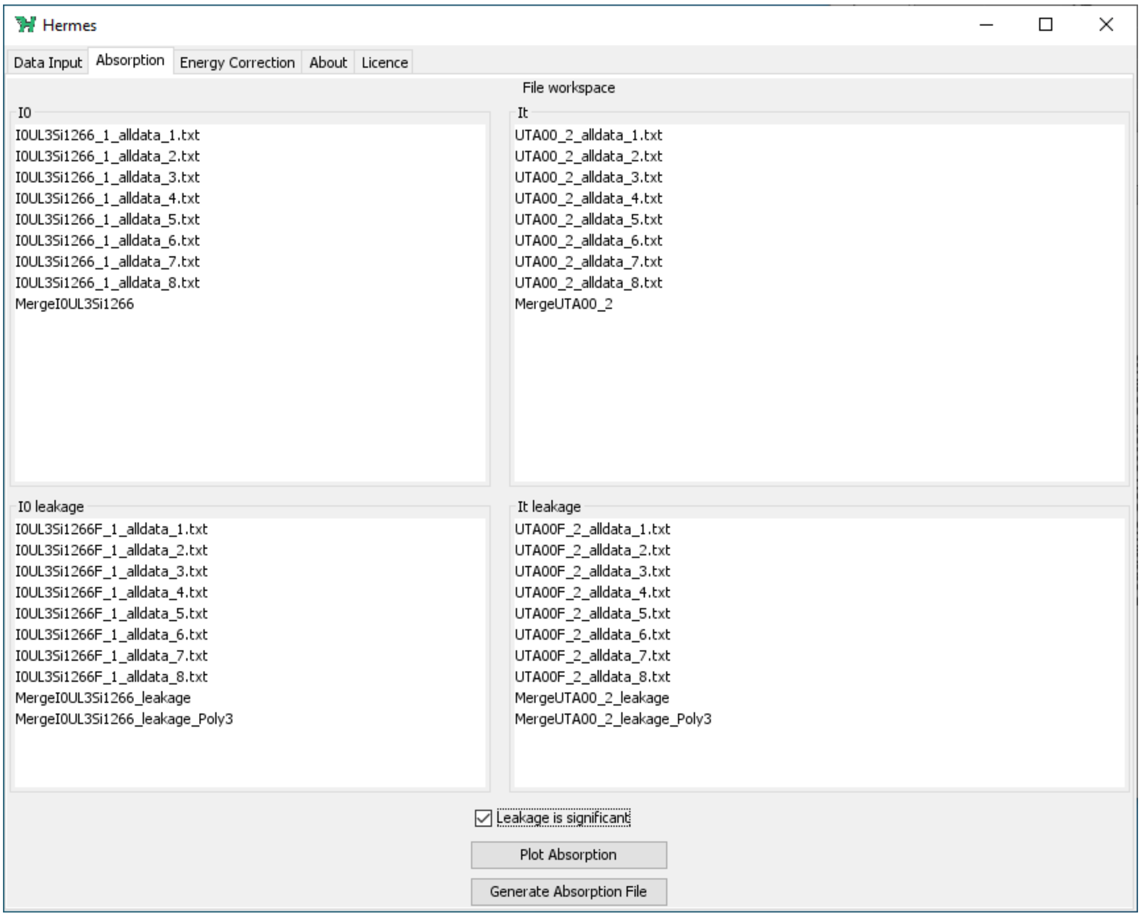

Figure 8: HERMES dashboard showing the Absorption tab, with four file workspaces (the **Leakage is significant** check box is ticked), and data processing and plotting tools.

6.1 Plot absorption

To evaluate and plot absorption data, first choose the constituent data within the designated workspace by clicking to highlight the data files. Select either two files (one each of I_0 and I_t), or four files if a leakage correction is required (one each of I_0, I_t, I_0 leakage, I_t leakage). The selected data may be raw, merged or polynomial fit data. Clicking **Plot absorption** will display the computed absorption as both a function of Energy (eV) and Theta (degrees), as shown in Figure 9. The plot may be operated on as described above. If the constituent data are not of the same range, the user will be notified with an error message and HERMES will truncate the output to the range of the shortest input data.

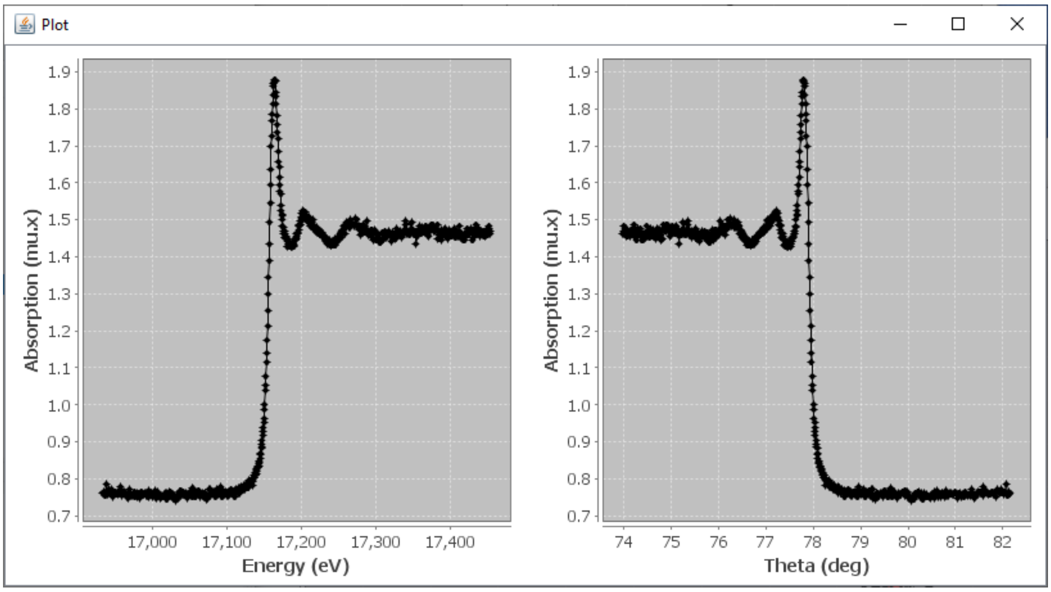

Figure 9: Example of absorption vs Energy (eV) and Theta (Degrees) plots, here generated with leakage correction from merged I_0 and I_t data, and polynomial fit to merged I_0 leakage and I_t leakage data.

6.2 Generate absorption file

Choose the constituent data to generate the absorption file by clicking to highlight the data files. Select either two files (one each of I_0 and I_t), or four files if a leakage correction is required (one each of I_0, I_t, I_0 leakage, I_t leakage). The selected data may be raw, merged or polynomial fit data. Click the **Generate Absorption file** button to write a file containing the computed absorption and constituent data (i.e. I_0, I_t, I_0 leakage, I_t leakage). Enter a filename and header for the new file in the dialogue boxes. The header can contain any information useful to the user, for example, relating to the sample composition or parameters of measurement. The absorption data file*, absorptionfilename.dat*, and an associated list file, *absorptionfilename.txt,* are written to a user specified folder in the subsequent dialogue box. If the constituent data are not of the same range, the user will be notified with an error message and HERMES will truncate the output data file to the range of the shortest input file.

The format of the absorption data file is as a space delimited text file, with columns of:

1. Energy (eV)
2. Theta (degrees)
3. Absorption (µx)
4. I_0
5. I_t
6. I_0 – I_0 leakage
7. I_t – I_t leakage
8. I_0 leakage
9. I_t leakage

The absorption data file may be input to the ATHENA program, choosing the appropriate columns for Energy, I_0 and I_t, for further analysis in the normal way. The other columns are included to facilitate further analysis using one composite data file, for convenience.

The list file contains the file name and header line of each of the constituent data sets used to build the absorption file.

7. The Energy Correction tab

This tab facilitates calibration of the energy scale of laboratory XAS data. It features a single workspace which displays Absorption Files created in the Absorption tab in the current session, as shown in Figure 10.

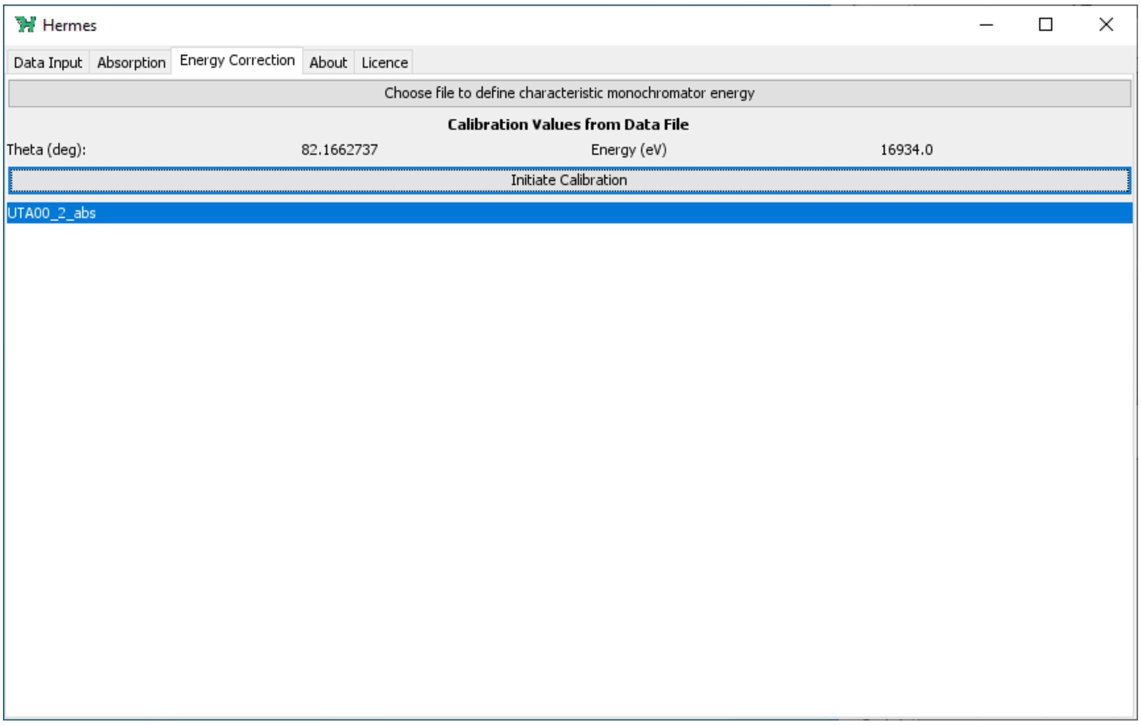

Figure 10: HERMES dashboard showing the Energy Correction tab, with one file workspace; the Absorption File was imported from the previous tab (corresponding to data shown in Figure 9).

Rowland circle spectrometers function on an angle dispersive principle to maintain the required focussing arrangement. Constant steps (i) in energy or k- space, as user specified ranges, determine the required steps in theta space according to the Bragg Law:

$$E_{mono} = E_i \sin\theta_i \qquad \text{Eq. 3}$$

Where E_{mono} is the characteristic backscatter energy of the monochromator.

Laboratory XAS data typically require calibration of the energy scale for comparison with synchrotron or other data. This can be achieved by acquisition of data from a reference material, for example – a metal foil, for which the true energy value of some feature in the data is known, such as the maximum in the first derivative of μ(E). Since the relationship between energy and theta is non-linear, it is necessary to apply the correction in theta space and then recalibrate the energy scale. The following method is implemented by HERMES:

- The appropriate E_{mono} is determined by picking the first pair of E_i and θ_i values from a user specified data set (Equation 3).
- The user specifies the observed (E_{obs}) and true (E_{true}) energy of some feature in the data (in eV), for example the maximum in the first derivative of μ(E).

- The corresponding θ_{obs} and θ_{true} values are determined (Equation 4 and 5) and the shift required to correct the θ_{obs} values (Equation 6); for information, in working with trigonometric functions HERMES converts between degrees and radians, under the hood.

$$\theta_{obs} = \sin^{-1}\left(\frac{E_{obs}}{E_{mono}}\right) \qquad \text{Eq. 4}$$

$$\theta_{true} = \sin^{-1}\left(\frac{E_{true}}{E_{mono}}\right) \qquad \text{Eq. 5}$$

$$\theta_{shift} = \theta_{true} - \theta_{obs} \qquad \text{Eq. 6}$$

- The calibration procedure then corrects the θ_{obs} values by adding the calculated θ_{shift} (Equation 6) and determines the corresponding $E_{correct}$ value according to Equation 7:

$$E_{correct} = \left(\frac{E_{mono}}{\sin(\theta_{obs} + \theta_{shift})}\right) \qquad \text{Eq. 7}$$

7.1 Choose file to define characteristic monochromator energy

The file workspace displays the Absorption Files previously created during the session, in the Absorption tab. Exactly one Absorption file must be selected by the user to extract an E_i and associated θ_i value to compute E_{mono}, as discussed above. This is initiated by first highlighting the desired Absorption file and then clicking **Choose file to define characteristic monochromator energy**. HERMES will then display the extracted E_i and θ_i values, and compute E_{mono}, as shown in Figure 10.

7.2 Initiate calibration

To correct the energy scale of an Absorption File, HERMES requires the user to input the observed energy and true energy of some feature in the data, for example the maximum in the first derivative of μ(E). These energies can be determined by loading the Absorption File into ATHENA and comparison to a reference or literature data set.

Click **Initiate calibration** to start the recalibration process. HERMES will first display the computed E_{mono} value for acceptance by the user, then request the observed energy and true energy of some feature in the data to be entered into the subsequent dialogue boxes. The user is then asked to specify the folder to which the corrected Absorption file should be saved as *absorptionfilename_corr.dat*. Finally, the original and corrected absorption data are shown as an overlay plot, in Energy and Theta space, as shown in Figure 11. The plot may be operated on as described above.

The format of the absorption data file is as a space delimited text file, with columns of:

1. Energy (eV)
2. Theta (degrees)
3. Absorption (μx)
4. I_0
5. I_t
6. I_0 – I_0 leakage
7. I_t – I_t leakage
8. I_0 leakage
9. I_t leakage
10. Corrected Energy (eV)
11. Corrected Theta (degrees)

The list file captures the relevant calibration data, as summarised below, with parameters for the calibration shown in Figure 10.

- Calibration data file name: UTA00_2_abs
- Calibration Energy (eV): 16934.0
- Calibration Theta (deg): 82.1662737
- Emono (eV): 16775.968218697788
- Theta Shift (deg): -0.1808647122174706
- Observed Energy (eV): 17157.50 eV
- True Energy (eV): 17169.21 eV

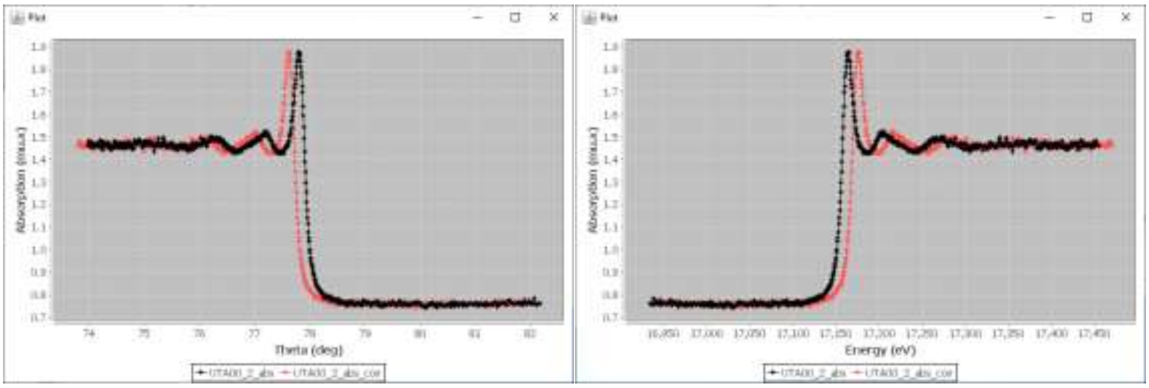

Figure 11: Showing original (black) and energy corrected (red) absorption data in left) theta space, and, right) energy space (corresponding to data shown in Figure 9). The calibration utilised E_{obs} = 17157.50 eV and E_{true} = 17169.21 eV

8. The About tab

This tab provides information on the version of HERMES software, its development, and funding sources.

9. The License tab

This tab provides information on the MIT software licence under which HERMES is made available.

10. Resources

A project page for HERMES exists at https://github.com/xasheffield/hermes and https://sourceforge.net/projects/hermes-xa-sheffield/. HERMES is available as an executable jar file (Windows, MacOS, requiring an existing installation of Java) via the Github link or as an executable file with the necessary Java Runtime bundled (Windows only) via the Sourceforge link, and both are freely available at the web page mentioned above, as well as a complete user manual and tutorial video.

Acknowledgements

The HERMES software was developed with resources of EPSRC Impact Acceleration Account at the University of Sheffield under grant EP/R511754/1 and the HADES/MIDAS facility at the University of Sheffield established with financial support from EPSRC and BEIS, under grant EP/T011424/1 [14]. The procedure for determination of theta shift was originally established by D. Mortensen.

References

1. G.T. Seidler, D.R. Mortensen, A.J. Remesnik, J.I. Pacold, N.A. Ball, N. Barry, M. Styczinski, O.R. Hoidn, Rev. Sci. Instr., 85, 113906 (2014).
2. D.R. Mortensen, G.T. Seidler, A.S. Ditter, P. Glatzel, J. Phys. Conf. Ser., 712, 012036 (2016).
3. G.T. Seidler, A.S. Ditter, N.A. Ball, A.J. Remesnik, J. Phys. Conf. Ser., 712, 012015 (2016).
4. E.P. Jahrman, W.M. Holden, A.S. Ditter, D.R. Mortensen, G.T. Seidler, T.T. Fister, S.A. Kozimor, L.F.J. Piper, J. Rana, N.C. Hyatt, M.C. Stennett, Rev. Sci. Instr., 90, 024106 (2019).
5. A.P. Honkanen, S. Ollikkala, T. Ahopelto, A.J. Kallio, M. Blomberg, S. Huotari, Rev. Sci. Instr., 90, 033107 (2019).
6. L.M. Mottram, S. Cafferkey, A.R. Mason, T. Oulton, S.K. Sun, D.J. Bailey, M.C. Stennett, N.C. Hyatt, J. Geosci., 65, 27 (2020).
7. B. Ravel, M. Newville, J. Synch. Rad., 12, 537 (2005).
8. The MIT Licence, Open Source Initiative, https://opensource.org/licenses/MIT
9. Creative Commons, Attribution 4.0 International licence (CC BY 4.0) https://creativecommons.org/licenses/by/4.0/
10. JFreeChart, https://www.jfree.org/jfreechart/
11. microDXP Technical Reference Manual v1.1.2 - XIA LLC https://xia.com/wp-content/uploads/2018/04/microDXP_Technical_Reference_Manual_v1.1.2.pdf
12. E. A. Stern and K. Kim, Phys. Rev. B, 23, 3781 (1981).
13. L.M. Mottram, M.C. Dixon Wilkins, L.R. Blackburn, T. Oulton, M.C. Stennett, S.K. Sun, C.L. Corkhill, N.C. Hyatt, MRS Adv., 5, 37 (2020).
14. N.C. Hyatt, C.L. Corkhill, M.C. Stennett, R.J. Hand, L.J. Gardner, C.L. Thorpe, IOP Conf. Ser. Mater. Sci. Eng. 2020, 818, 012022.
